# Supplementary material for: Dynamical state of the network determines the efficacy of single neuron properties in shaping the network activity
Source: Sci Rep. 2016 May 23;6:26029. doi: 10.1038/srep26029 (PMC4876512; doi:10.1038/srep26029)
Supplement: Supplementary Information [file srep26029-s1.pdf]

Dynamical state of the network determines the efficacy of single neuron properties in shaping the network activity

Ajith Sahasranamam[1,2], Ioannis Vlachos[1,2], Ad Aertsen[1,2], and Arvind Kumar[1,3,*]

[1]Bernstein Center Freiburg, University of Freiburg, 79104 Freiburg, Germany.

[2]Faculty of Biology, University of Freiburg, 79104 Freiburg, Germany.

[3]Dept. of Computational Science and Technology, School of Computer Science and Communication, KTH Royal Institute of Technology, Stockholm, 10044, Sweden

ABSTRACT

Here we have provided additional figures to support our results and main conclusions.

Supplementary figures

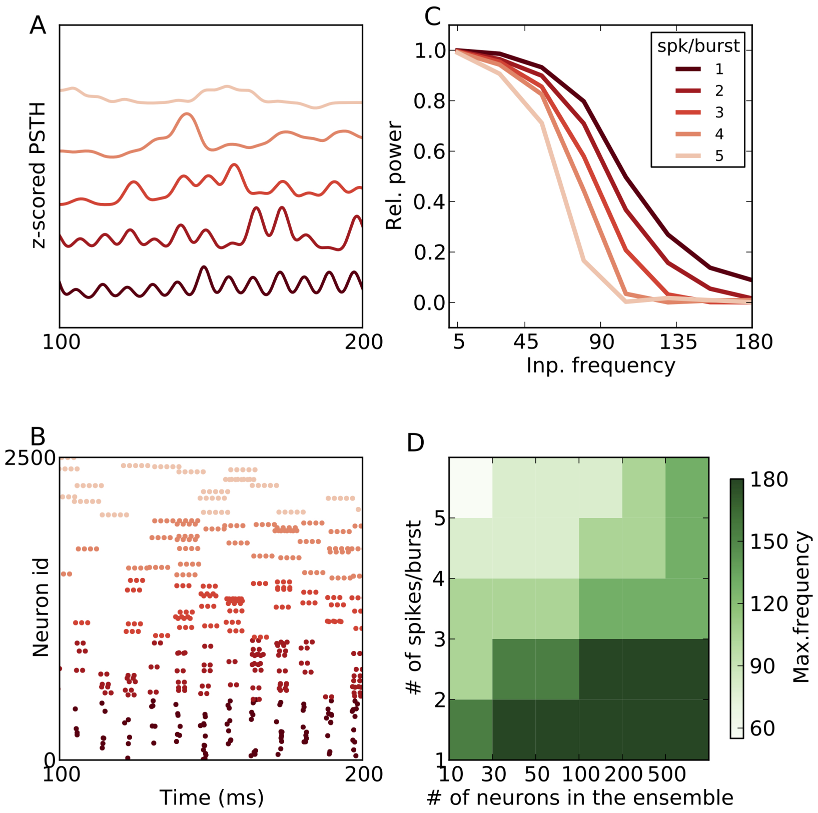

Figure 1. Increased bursting reduces the frequency of input oscillations that can be tracked. In unconnected networks of different number of modified-SSBNs, we test how well sinusoidally modulated input of different frequencies could be followed by the population. For higher input frequencies, it is seen that for increased number of spikes per burst are less able to follow the input. The rasters and the z-scored PSTHs for different number of spikes per burst for a fixed sinusoidal input (120 Hz) are shown in (**A**) and (**B**). (**C**) For a fixed size of the neuron ensemble ($N = 100$) the normalized power of the peak frequency drops and saturates to a very small value (≈ 0) for higher frequencies of the sinusoidally modulated input. (**D**) The map shows the maximum frequency of the input that can be tracked by different combinations of number of independent neurons in the population and the number of spikes per burst. While the value of the frequency drops with the increase in the number of spikes per burst, it can be compensated for by increasing the number of neurons in the ensemble.

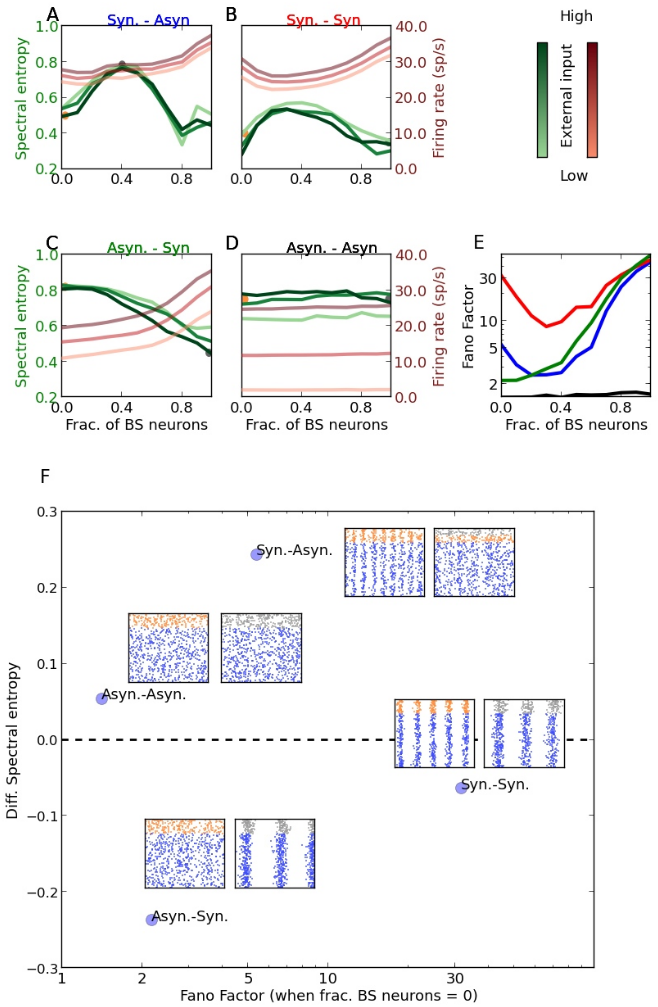

Figure 2. Figure caption continued on the following page

Figure 2. Effect of addition of bursting neurons on the state of network composed of SSBN. (**A**) Evolution of spectral entropy(H_S) for a network which is initially synchronous and changes to being asynchronous with the addition of bursting neurons,added ($g = 11, d = 2ms, \eta = 10500 - 11500\, sp/s, J_E = 0.1\, mV$).(**B**) a network in an asynchronous state that continues to remain asynchronous with the addition of bursting neurons ($g = 5, d = 2.0ms, \eta = 4000 - 5000 sp/s, J_E = 0.04 mV$) , **C** qualitatively synchronous activity can remain synchronous even when inhibitory neuron firing patterns are changed ($g = 8, d = 4ms, \eta = 8500 - 9500 sp/s, J_E = 0.1\, mV$) and (**D**) an initially asynchronous activity in the network that becomes synchronous with the addition of bursting neurons ($g = 6, d = 2ms, \eta = 4500 - 5500 sp/s, J_E = 0.1\, mV$). (**E**) The Fano Factor values of the different transitions are plotted against the changes in the fraction of bursting neurons. The different colours correspond to the different state transitions observed (colours marked in the titles of **A**,**B**,**C** and **D**). (**F**) The rasters illustrating the four types of transitions are shown in a phase space of FF and the difference in H_S. The difference in H_S is the difference in spectral entropy between the initial and final points of each transitions.The initial rasters are marked in yellow and the final rasters are marked in black in the corresponding panels **A**,**B**,**C** and **D**. The FF values marked are the FF values of the initial points.

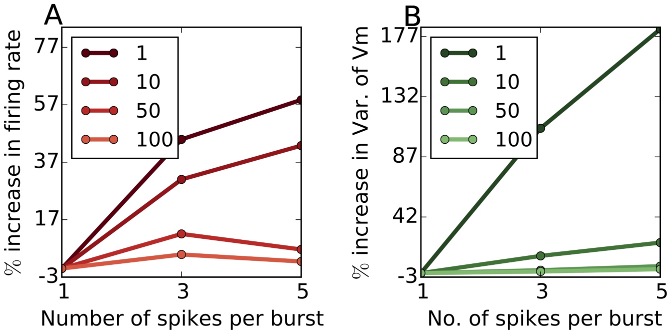

Figure 3. A simple network producing an external input induced spiking of a presynaptic BS population. This BS population acted as the inhibitory presynaptic input to a regular LIF neuron. The membrane potential of this LIF neuron was maintained very close to the threshold by an external poissonian input. The percentage change in the variance of the membrane potential (**A**) and firing rate (**B**) of the postsynaptic LIF neuron with the varying number of spikes per burst in the presynaptic SSBN population is plotted. The increase in the size of the presynaptic population decreased the amount of changes in the variance of the membrane potential and the firing rate of the post-synaptic LIF with the change in the number of spikes per burst.

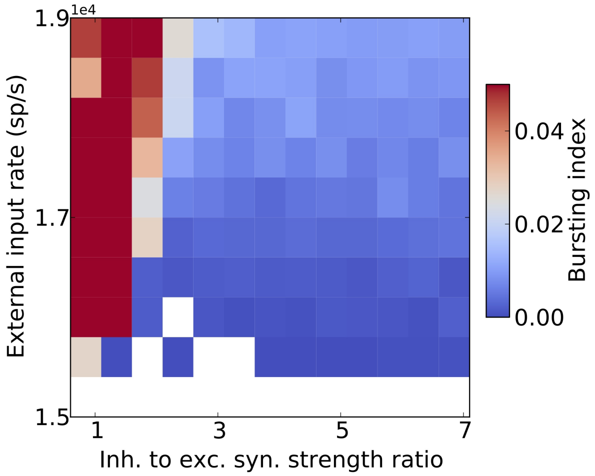

Figure 4. Burstiness of single neurons changes with network state. The number of spikes per burst that a BS neuron(Izhikevich model) produces depends on the state of the network. To quantify the burstiness of a neuron we use the Bursting Index[1] This measure assigns a rank R_n to every interspike interval (ISI) of a spike train. The lowest value of an ISI has zero rank. If the ISIs are independent, the value of each ISI can be considered to be a random number drawn from a uniform distribution between 1 and N, where N is the total number of ISIs. If a spike train contains a burst, then this assumption does not hold anymore. The Bursting Index is equivalent to the Rank Surprise (RS) statistic, which captures the discrepancy between the case of having independent and uniformly distributed sequence of variables $R_n, ..., R_{n+q-1}$ and the actual outcome in the case of a burst consisting of q number of spikes. It is given given by $RS = -log(P(T_q \leq r_n + ... + r_{n+q-1}))$ where r_n is the observed value of rank R_n. T_q is the sum of q discrete uniform variates between 1 and N. In the above figure, the average bursting index of BS neurons for different η and g values are shown in a randomly connected network of excitatory-BS neurons (Izhikevich model).

References

1. Gourévitch, B. & Eggermont, J. J. A nonparametric approach for detection of bursts in spike trains. *J Neurosci Methods* **160**, 349–58 (2007).
